# Estimation of the concentrations of hydroxylated polychlorinated biphenyls in human serum using ionization efficiency prediction for electrospray

**DOI:** 10.1007/s00216-022-04096-2

**Published:** 2022-05-04

**Authors:** Sara Khabazbashi, Josefin Engelhardt, Claudia Möckel, Jana Weiss, Anneli Kruve

**Affiliations:** 1 Department of Materials and Environmental Science, Stockholm University, Svante Arrhenius väg 16, 106 91 Stockholm, Sweden; 2 Department of Environmental Science, Stockholm University, Svante Arrhenius väg 8, 106 91 Stockholm, Sweden

**Keywords:** Non-targeted screening, Machine learning, Matrix effect, Ionization efficiency, Quantification, LC/HRMS

## Abstract

Hydroxylated PCBs are an important class of metabolites of the widely distributed environmental contaminants polychlorinated biphenyls (PCBs). However, the absence of authentic standards is often a limitation when subject to detection, identification, and quantification. Recently, new strategies to quantify compounds detected with non-targeted LC/ESI/HRMS based on predicted ionization efficiency values have emerged. Here, we evaluate the impact of chemical space coverage and sample matrix on the accuracy of ionization efficiency-based quantification. We show that extending the chemical space of interest is crucial in improving the performance of quantification. Therefore, we extend the ionization efficiency-based quantification approach to hydroxylated PCBs in serum samples with a retraining approach that involves 14 OH-PCBs and validate it with an additional four OH-PCBs. The predicted and measured ionization efficiency values of the OH-PCBs agreed within the mean error of 2.1 × and enabled quantification with the mean error of 4.4 × or better. We observed that the error mostly arose from the ionization efficiency predictions and the impact of matrix effects was of less importance, varying from 37 to 165%. The results show that there is potential for predictive machine learning models for quantification even in very complex matrices such as serum. Further, retraining the already developed models provides a timely and cost-effective solution for extending the chemical space of the application area.

**Supplementary Information:**

The online version contains supplementary material available at 10.1007/s00216-022-04096-2.

## Introduction

Polychlorinated biphenyls (PCBs) have been produced on a large scale until their regulation in the 1970s due to the discovery of their negative human and environmental effects [1]. PCBs are persistent and show strong resistance towards chemical and biological degradation; therefore, they are still prevalent pollutants in the environment [1]. In spite of their stability, PCBs can degrade and metabolize. For example, hydroxylated polychlorinated biphenyls (OH-PCBs) are produced in the environment by oxidative transformation through metabolic and abiotic processes and are the most commonly reported PCB metabolites in human blood. Approximately 40 OH-PCBs have been identified in human blood [1]. Compared to their parent congeners, the addition of a hydroxyl group in the OH-PCBs reduces volatility and increases hydrophilic properties and water solubility [2]. However, the compounds largely retain their hydrophobic character (log*P* ≈ 5) due to the presence of chlorinated phenyl rings [3]. In contrast to PCBs, OH-PCBs are weak acids which facilitate the analyzability with liquid chromatography electrospray mass spectrometry (LC/ESI/MS) [4].

The identification and quantification of OH-PCBs are largely limited by the availability of analytical standards. Recently, different strategies to quantify contaminants detected with non-targeted screening with liquid chromatography electrospray high resolution mass spectrometry (LC/ESI/HRMS) have been proposed [5, 6]. One of the simplest approaches suggests the usage of the calibration graph of the parent compounds for quantification of the metabolites and transformation products. Unfortunately, in case of LC/HRMS analysis of OH-PCBs, the parents, PCBs, are not detectable with the same analytical methods. Alternatively, we have recently proposed that the ionization efficiency of contaminants in ESI/HRMS can be predicted with machine learning and used thereafter for quantification of these chemicals. Promising results have been obtained for the quantification of contaminants in water[7, 8] and food[9] in ESI positive mode. The prediction errors obtained have ranged from 1.7 × to 5 × depending on compound and matrix complexity. The applicability for more complex tasks such as exposomics has not yet been reviewed.

The accuracy of the prediction-based quantifications depends on (1) the model and (2) other factors affecting measurement quality, such as matrix effects. The model prediction accuracy is largely affected by the overlap of the chemical space of interest with the chemical space covered by the compounds used in model training. Until now, PCB metabolites and chemically similar compounds have been strongly underrepresented in the chemical modelling datasets due to the lack of analytical standards. Additionally, losses in sample preparation [5] may occur and complex sample matrices are known to cause matrix effect [10, 11] in the electrospray, which mostly yields ionization suppression and decreased signals in the presence of matrix components [10]. Coping with matrix effect is cumbersome in targeted analysis where isotopically labelled standards are most effective but sample dilution [12], extrapolative dilution [13], matrix matching, and (post-column) standard addition [14] have also been investigated. Recently, Tisler et al. [15] have proposed a three-stage approach for accounting for matrix effect in non-targeted screening which includes sample dilution, total-ion-count-based correction, and quantitative structure property relationship (QSPR) modelling of matrix effect. Therefore, the quantification of PCB metabolites in non-targeted LC/ESI/HRMS analysis requires overcoming the burden of restricted chemical space and ionization suppression occurring in ESI.

Here, we will investigate the impact of chemical space coverage of the machine learning models for predicting the ionization efficiency (IE) values on the example of OH-PCBs. Thereafter, we will widen the chemical space coverage for the quantification of OH-PCBs in negative mode LC/ESI/HRMS suspect analysis. We hypothesize that retraining the existing models with chemicals with sufficiently similar chemical properties and thus expanding the chemical space will significantly improve the prediction accuracy. Last but not least, we evaluate the impact of matrix effect on the quantification accuracy.

## Materials and methods

### Chemicals

HPLC grade methanol (≥ 99.9%, RiedeldeHaen, Honeywell, Seelze, Germany, 67–56-1) was used for sample preparation. HPLC gradient grade acetonitrile (≥ 99.9%, RiedeldeHaen, Honeywell, Seelze, Germany, 75–05-8) was used for sample preparation and as organic modifier. Ammonium acetate buffer was made from ammonium acetate (Sigma-Aldrich, Darmstadt, Germany, 631–61-8) and HPLC grade water (RiedeldeHaen, Honeywell, Seelze, Germany, 7732–18-5). Laboratory reagent grade hydrochloric acid washed with DCM (32% J.T. Baker, Radnor, USA, 75–09-2), potassium hydroxide pellets (Merck, Darmstadt, Germany, 1310–58-3), 2-propanol for gas chromatography ECD and FID (Sigma-Aldrich, Darmstadt, Germany, 67–63-0), isohexane for HPLC (≥ 97.0%, VWR Chemicals, Leuven, Belgium, 92,112–69-1), and HPLC grade methyl tertbutyl ether (MTBE) (Rathburn Chemicals Ltd. Walkerburn, Scotland, 1634–04-4) were used for pretreatment of the human serum samples.

The OH-PCB standards have been synthesized in house (Department of Environmental Science, Stockholm University) over the years (method description is available from Bergman et al. [16]), contained in sealed ampoules with chloroform or 4-methyl-2-pentanone as solvent. Additionally, 2,3,5,6-tetrafluoro-4-(pentafluorophenyl)phenol and pentakis(trifluoromethyl)phenol were kind gifts from A. Kütt [17]. Perfluorooctane sulfonic acid (PFOS) was a kind gift of Prof. Taft. A total of 21 standards were acquired and described in more detail in Table S1.

### Sample preparation

The pretreatment was performed according to a large-scale procedure for analysis of phenolic substances in serum reported by Hovander et al. [18]. A detailed protocol of the work flow is given in the supporting information. In short, 50 mL human serum from 2 Swedish blood donors was combined in a beaker to give a pooled sample. The serum was transferred to a separation funnel and denatured with hydrochloric acid and isopropanol. The OH-PCBs were extracted using 1:1 isohexane/MTBE. The organic phase was washed with 1% KCl, and transferred to a round-bottom flask.

To separate the neutral and polar fraction, 1 M KOH was added and the funnel inverted 30 times. The phases were allowed to separate and the KOH fraction was collected. HCl (4 mL, 2 M) was added to acidify the hydroxyl groups. The phenolic compounds were extracted with 9:1 isohexane/MTBE. The sample was portioned into aliquots corresponding to 1.4 mL, 2 mL, and 4 mL of serum each, in triplicates. The procedure was duplicated in order to have an identical setup for samples fortified with the native OH-PCBs for quality control. Half of the samples were fortified with 100 µL of the standard mixtures (31 to 59 ng/mL) and an equivalent amount of methanol was added to the other half of the samples. All samples were evaporated to 100 µL before injection on the LC–MS system. The matrix effect was calculated as

1 $$\%ME=\frac{{c}_{detected}}{{c}_{added}}\bullet 100\%$$

where 100% indicates no matrix effect and values under 100% indicate ionization suppression.

### Instrumentation

All samples were analysed on an Acquity Ultra Performance Liquid Chromatograph (UPLC, Waters Corporations, MA, USA) coupled with a Waters Select Series cyclic ion mobility (cIM) mass spectrometer. Standard samples were injected in flow injection mode (FIA) and onto a Phenomenex Kinetex PS C18 LC column (2.6 µm, 3 × 150 mm) on the same chromatograph. Samples were injected onto the LC column in replicates (*n* = 3) and with varying injection volumes (see discussion below).

The mobile phase consisted of (A) 10 mM ammonium acetate buffer in water, pH = 8.0, and (B) acetonitrile. In flow injection mode, the mobile phase composition was 20% A and 80% B at a flow rate of 0.3 mL/min. The LC analysis was performed using a linear gradient elution at a flow rate of 0.35 mL/min at 30 °C. The gradient was from 10 to 90% B in 15 min, and then maintained at 90% B for 5 min and returned back to 10% B for 6 s. Allowing 3 min for equilibration with 10% B gave a total runtime of 23 min. Dwell volume was not taken into account. The responses of [M–H]^−^ were recorded in MS full scan negative mode conducted in the mass to charge (*m/z*) range between 50 and 1200 Da. The signals of the molecular peaks and any [M–HCl]^−^ and [M–H–HCl]^−^ insource fragments > 1% were summed and isotope corrected.

### Ionization efficiency measurements

Each measurement of the standards used for ionization efficiency measurements (8 to 15 ng/mL) was run in duplicates on two different days in flow injection analysis (FIA) and with LC with previously mentioned settings. Standard solutions were measured separately in flow injection mode in a continuous run. In LC mode, standards were measured in groups consisting of three to four compounds based on differences in molecular weight and diversity in molecular structure in order to ensure good separation. Calibration points were established by injecting nine different volumes of the standards with the autosampler (1 to 10 μL). 2,3,5,6-Tetrafluoro-4-(pentafluorophenyl)phenol, pentakis(trifluoromethyl)-phenol, and perfluorooctane sulfonic acid were measured in all of measurements on each day. Calibration curves for every compound measured in flow injection analysis and with LC were constructed. The slope of the signal versus concentration calibration graphs was estimated via linear regression in the linear range. If the intercept was statistically insignificant, intercept was set to zero. The slope was then corrected with the relative abundance of the isotope correction factor (*IC*). Relative ionization efficiency (*RIE*) values were calculated relative to so-called anchore compound: 2,3,5,6-tetrafluoro-4-(pentafluorophenyl)phenol, pentakis(trifluoromethyl)phenol, and perfluorooctane sulfonic acid, for which IE values have been measured previously [19]. *RIE* for a compound (*M*_1_) was calculated relative to the anchor compound (*M*_2_) according to Eq. 2.

2 $$RIE\left({M}_{1}/{M}_{2}\right)=\frac{slope\left({M}_{1}\right)\bullet IC\left({M}_{1}\right)}{slope\left({M}_{2}\right)\bullet IC\left({M}_{2}\right)}$$

The log*IE* values were calculated based on log*RIE* and previously measured [19] ionization value of the anchor compound.

3 $${\mathrm{Log}IE}_{{M}_{1}}=\mathrm{log}RIE\left({M}_{1}/{M}_{anchor}\right)+{\mathrm{log}IE}_{anchor}$$

### Ionization efficiency prediction model

Firstly, the previously developed model from Liigand et al.[20] was evaluated for predicting the log*IE* of OH-PCBs. To describe the structure of the chemicals, 1444 PaDEL descriptors [21] were calculated for all OH-PCBs. PaDEL descriptors capture fingerprint type of descriptors, such as number of carbon, oxygen, halogen atoms present or presence of alcohol group, amine group, as well as topological (volume, longest aliphatic chain, length over breadth) and polarity (*X*log*P*, polar surface area, number of given and accepted hydrogen bonds, etc.) related fingerprints. Additionally, five eluent descriptors, namely viscosity, polarity index, surface tension, pH of the water phase, and NH_4_^+^ content (“yes” or “no”), were used. log*IE* values for all OH-PCBs analysed both in FIA and LC were predicted.

In order to improve the predictive power for PCB metabolites, a wider scope model was trained. Previously measured log*IE* values in negative mode presented in the research of Kruve et al.[22] and Liigand et al.[20] were used as a base. The log*IE* values came from 100 unique compounds and 33 eluent compositions. Combined with the measurements of the OH-PCBs, a total of 118 unique compounds, 35 eluent compositions, and 1328 log*IE* values have been acquired.

For every compound, 1460 PaDEL descriptors as well as eluent descriptors were calculated and added to the dataset. Descriptors for which the frequency of the most common value was above 95%, i.e. near zero variance, were removed from the dataset. To reduce the overfitting, highly correlated descriptors were removed by checking a pairwise correlation between the descriptors and keeping only the descriptors with *R*^2^ < 90%. This resulted in a total of 406 descriptors in the final dataset. The dataset was divided into OH-PCBs and previously measured compounds. Both sets were shuffled and split into (1) training set accounting for 80% of the studied chemicals and (2) test set accounting for the remaining 20% of the chemicals. The training sets were then concatenated, as were the two test sets. The training set was used for training the models while the test set was used for evaluating the model’s generalization capabilities. Several machine learning algorithms were tested in the model development where suitable R packages were leveraged. These included support vector machine regression (*svmLinear*), random forest regression (*rf*), regularized random forest (*RRF*), quantile random forest regression (*qrf*), and eXtreme Gradient Boosting (*xgbTree* and *xgbLinear*). The final model was trained with *xgbTree*.

The similarity between OH-PCBs and the previously published data[20] was calculated as cosine similarity over all calculated PaDEL descriptors.

## Results and discussion

### Ionization efficiency values

The 18 OH-PCBs showed a large variability in log*IE* values for measurements with both FIA (one mobile phase composition) and LC separation; all values can be found in Table S1. For flow injection analysis, the lowest log*IE* of 1.94 was observed for 4′-OH-CB30 and the highest of 3.29 was observed for 4-OH-CB127, while with LC separation the log*IE* of 2.17 was observed for 4-OH-CB107 and highest value of 3.41 for 4-OH-CB127. In general, the log*IE* values increased with the degree of chlorination and molecular weight (see Figs. S1 and S2). This is also expected as both are in correlation with increased hydrophobicity for OH-PCBs and it is generally known that compounds with larger hydrophobic moieties are more easily moving to the surface of the ESI droplets [23], which increases the log*IE*. Within isomeric compounds, the log*IE* values varied up to 1.35 logarithmic units (22.4x) for tetrachlorinated OH-PCBs though predicted log*P* values showed minimal differences. In this group, significantly higher log*IE* values were observed for the 2-OH-PCB vs 4-OH-PCB. Significant differences in the ionization efficiency of *ortho*- and *para*-isomers of substituted phenols and benzoic acids have been observed previously [19], though in these cases 2-phenylphenol yielded slightly lower log*IE* values than did 4-phenylphenol. For dihydroxylated PCBs, the differences in log*IE* values were less significant, at maximum 6.3x.

The log*IE* values measured with flow injection analysis and LC separation were in weak correlation with *R*^2^ of 0.56 (see Fig. S3). The differences in log*IE* values can be related to the change in the organic modifier content at the time of elution. In case of FIA, all compounds were introduced to the ESI source in 80/20 mixture of acetonitrile/buffer while in LC analysis compounds eluted between 48 and 77% of acetonitrile. It was observed that for early eluting compounds the log*IE* values were mostly lower in LC analysis (see Fig. S4). This is expected as these compounds elute with less than 80% of organic modifier in the mobile phase and it is known that increase in organic modifier contentment boosts the ionization efficiency [24, 25]. Therefore, the structural effects as well as mobile phase effect observed for OH-PCBs agree with the previous finding.

### Evaluation of the previous model for log*IE* prediction

**Fig. 1:**
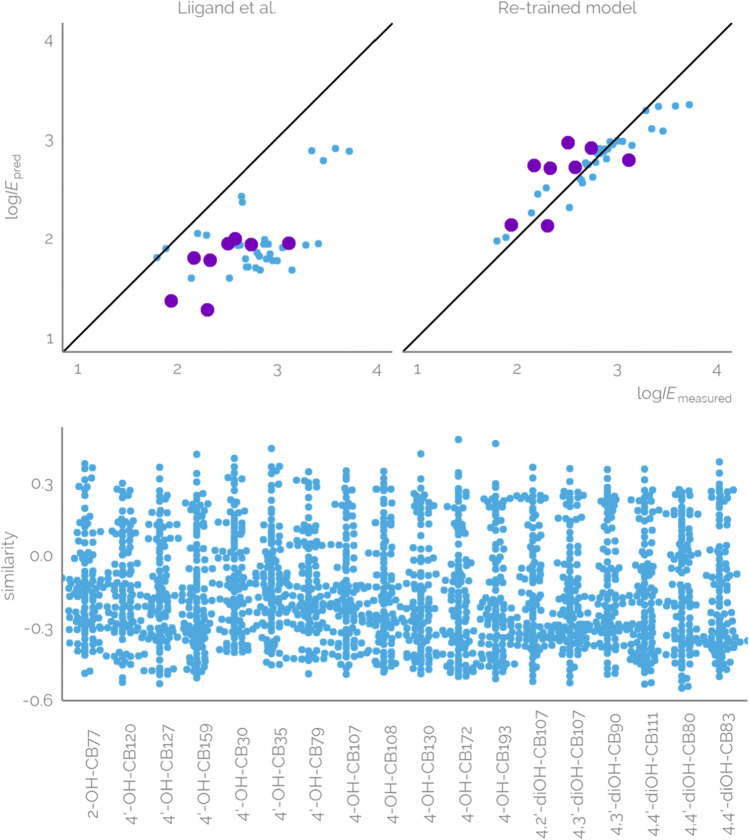
**a** The log*IE* prediction accuracy for OH-PCBs with previously trained model by Liigand et al.[20] and with the updated retrained model. For the updated model, the data added to the model are shown in blue and the OH-PCBs in the test set are shown in violet. **b** A bee swarm plot of the cosine similarity of the OH-PCBs to the dataset used for original model training by Liigand et al. [20]

Inspired by a good qualitative agreement in the log*IE* trends, it was of interest to evaluate how well a previously trained model can predict log*IE* values for a new compound group. For this, a previously trained random forest model by Liigand et al. [20] was used to predict the log*IE* values for all 18 OH-PCBs analysed with both FIA and LC. The predicted log*IE* values ranged from 1.36 to 1.98 for FIA and 1.27 to 2.04 for LC analysis. The predicted and measured log*IE* values for FIA showed a low but significant correlation with *R*^2^ of 0.53 while LC measurements showed a non-existing correlation with *R*^2^ of 0.01 (see Fig. 1). Also, the predicted log*IE* values were significantly lower than the measured values. Still, the predictions followed the observed increasing trend in log*IE* of OH-PCBs with increasing level of chlorination, indicating that a previously trained general model has learned some properties that also affect the ionization of OH-PCBs.

The predicted and measured ionization efficiency values may differ due to either (1) experimental differences or (2) insufficient model. The experimental differences may arise from significant alteration in the ionization mechanism with instrumental setup or overlooking some fragments formed from the deprotonated molecules during ionization. The insufficient model may, on the other hand, be generally trained on too few datapoints, with undescriptive features, or simply cover a different chemical space. The first two reasons for insufficient model would yield poor predictive power on any dataset and can be pinpointed during cross-validation and testing of the model. Therefore, significant gaps in predictive power of the pretrained model for OH-PCBs may hint towards insufficient coverage of chemical space by the training data. The compounds included in the training of the original model were a wide range of benzenoids, carboxylic acids, phenols, and sulfonamids. The eluent parameters varied with methanol or acetonitrile as organic modifier, content ranging from 0 to 100%; pH 2.78 to 10.5; water-phased additives ammonia, ammonium acetate, and formic acid ranging from 0 to 50 mM; and the presence of ammonium ions. Though the original dataset used for model training does incorporate some chlorinated phenols, chlorinated benzoic acids, and 4-(pentafluorophenyl)-2,3,5,6-tetrafluorophenol, a fully fluorinated analogue for 4-OH-PCB, the number of such compounds is low and possibly insufficient to learn the driving ionization mechanism for OH-PCBs.

The hypothesis of insufficient coverage of chemical space was also supported by the fairly low cosine similarity of the PaDEL descriptors of OH-PCBs and the training compounds for the pretrained model. The maximum similarities ranged between 0.27 and 0.48 depending on the OH-PCB and mean similarities ranged between − 0.09 and − 0.17 while median similarities ranged − 0.12 and − 0.24 (see Fig. 1). Here, 1.0 is an ideal similarity, 0 indicates no similarity, and − 1.0 indicates maximal dissimilarity. Therefore, the similarity between the OH-PCBs and the original training set is rather low and is likely to impact the low predictive power of the model for OH-PCBs.

It was additionally of interest if cosine similarity can be related to the prediction quality for individuals OH-PCBs. It was observed that OH-PCBs that had lower 95 percentile cosine similarity to the training data yielded a higher prediction error; however, the relationship was rather indicative then quantitative (see Fig. S4).

### Retraining the log*IE* prediction model

In order to improve the learning power of the model for OH-PCBs, the log*IE* predictive model was retrained. It is important that a sufficient number of OH-PCBs is seen by the model during training step while a sufficient number also remains for model evaluation. Therefore, we split the previously existing dataset of 100 unique compounds and the dataset collected in this study, 18 compounds, into both training and test sets with a 80:20 ratio. The training sets were combined into a single set and used for training a gradient boosted regression trees (*xgbTree*) model with fivefold cross-validation.

The test set compounds were left for evaluating the model. Overall, the root mean square error (RMSE) was 0.74 logarithmic units (5.5x) for the training set and 0.33 logarithmic units (2.1x) for the OH-PCBs in the test set. Therefore, the retraining of the model significantly improved the performance, from virtually no correlation for OH-PCBs analysed with LC before retraining to a high correlation of *R*^2^ of 0.82 after retraining (Fig. 1). This indicates a significant improvement in the model performance with the improved coverage of chemical space. The improved prediction accuracy can be related to the improved similarity between compounds in the test and training sets. The maximum cosine similarities increased from 0.48 to 0.96 for test set compounds.

The analysis of the descriptor importance for the old and new models indicates that the types of the majority of the top #20 descriptors in both of the models were the same. Though, the exact descriptors and importance of the descriptors, measured as total decrease in node impurity, changed between the models. In both models, some of the most important descriptors were autocorrelation parameters of mass and van der Waals volumes. Also, both models had mobile phase characteristics as important features in the model (see Table S2 and S3). This indicates that the general structural properties driving the ionization process and learned by the random forest algorithm are the same for both datasets. Still retraining with new compounds adjusts the model’s performance for the chemical space of interest.

### How many standards are needed?

It was also of interest to understand how the proportion of similar compounds in the training set influences the prediction accuracy for OH-PCBs. To evaluate this, two different starting sizes of the original training set were used, one containing 80 and one containing 40 unique compounds. We trained a number of gradient boosted regression trees by adding an increasing number of OH-PCBs to these two training sets. The OH-PCBs added to the training set were sampled randomly 15 times for each proportion and a new model was trained. The test set was fixed and contained in total 24 compounds, including four OH-PCBs while none of the test set compounds was in the training set.

**Fig. 2:**
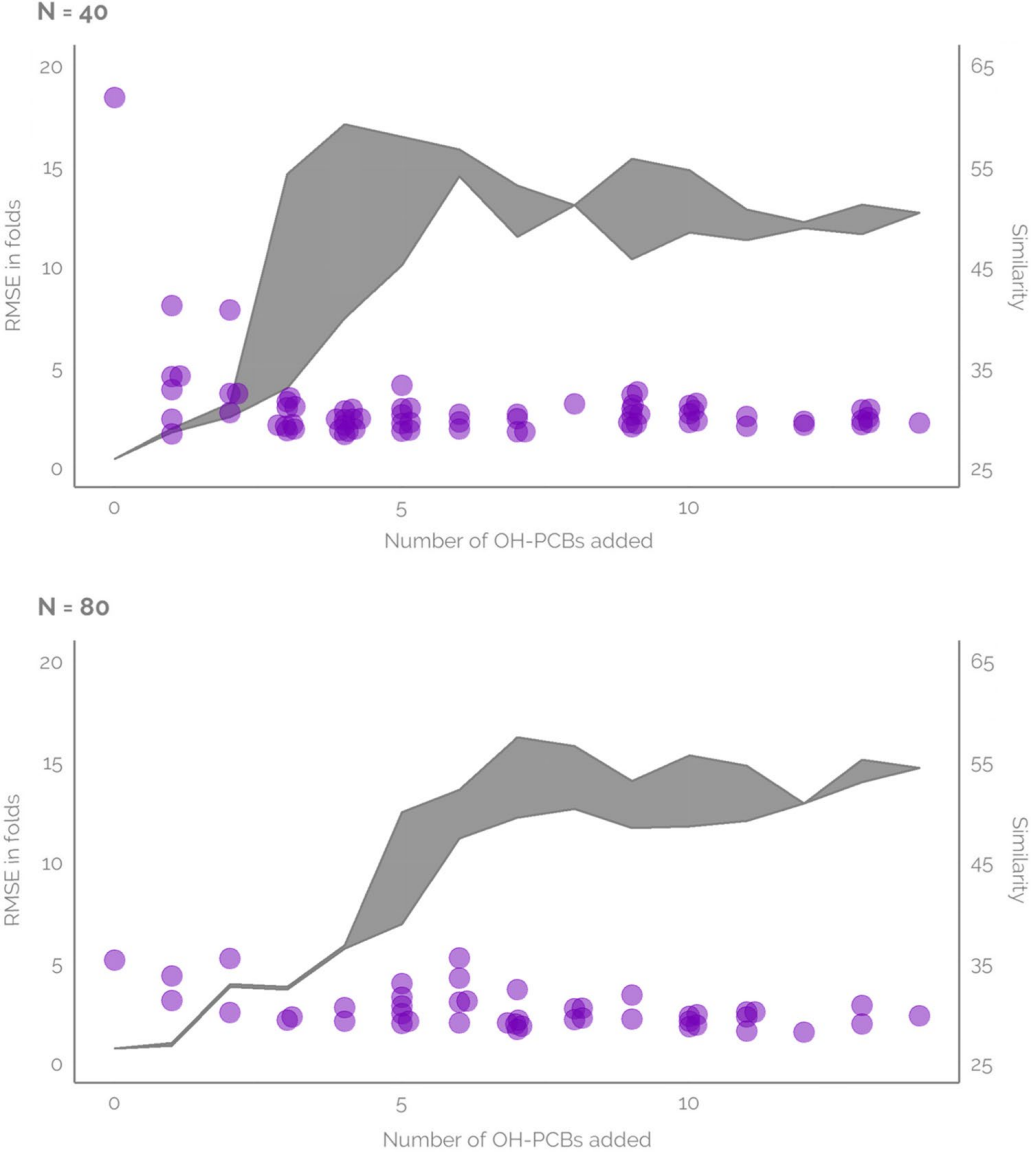
The root mean square prediction error for OH-PCBs (purple dots) depending on the number of OH-PCBs added to the model training set. The grey ribbon shows the 95 percentile cosine similarity between the OH-PCBs in the test set and all compounds in the training set. The upper and lower bounds of the ribbon show the highest and lowest similarities depending on the sampling of the OH-PCBs to the training set. The upper panel and lower panel differ in the number, *N* = 40 and *N* = 80, of the starting training set taken as the bases before adding any OH-PCBs

The models yielded RMSE of 0.18 to 1.26 logarithmic units (1.5x to 18.4x) on the OH-PCBs in the test set depending on the training set (see Fig. 2). It was observed that the addition of the OH-PCB results did not affect the prediction accuracy for the compounds not belonging to the group of OH-PCBs. However, for OH-PCBs, it was observed that adding four or more congeners to the model training significantly improved the prediction accuracy (see Fig. 2). Moreover, in case of smaller number of added OH-PCBs, the prediction accuracy varied strongly from model to model depending on the exact added OH-PCB. This indicates that adding one or two similar chemicals is not sufficient to allow accurate predictions of a new chemical group. The high variability of RMSE values depending on the added OH-PCBs indicates that model performance depends on the exact compounds added to the training set not merely the number of them.

Additionally, the number of compounds in the starting dataset had a significant impact on the model performance. The RMSE values for the smaller dataset showed even higher values and variability if only a small number of OH-PCBs had been added. This outlines the importance of the base dataset: the model trained on a larger dataset may show good and stable performance already if a couple of OH-PCBs are added to the training set.

Evaluation of the similarity of the training set and OH-PCBs in the test set revealed a sharp increase with the addition of a first few OH-PCBs to the model training set. However, after five to six OH-PCBs, the 95 percentile cosine similarity reached a plateau for both smaller and larger starting datasets. This roughly coincides with the number of OH-PCBs that yield a stable low RMSE value. That indicates that evaluating the similarity of the chemicals is useful in evaluating the suitability of the training set for a particular purpose.

It is of interest if every new group of chemicals requires training a new model. Here, the performance of the model is significantly improved with the addition of just 15% of new datapoints while the data used for training previously are completely retained. We also see that keeping a large fraction of previously collected training data for new model training increases the stability of the predictions and reduces the RMSE. We believe that this comes from the fact that the underlying ionization mechanism for the compounds considered previously and included in this study is very similar and the retraining accounts for the extension of the range of structural descriptor values and their combinations. Therefore, developing one generic log*IE* prediction model as long as the ionization mechanism does not change is plausible and finetuning a model for a new compound group should rather include adding new training instances to existing models than training a completely new model without benefiting from the existing models.

In addition to expanding ionization efficiency predictive models for new groups of chemicals, it is also of interest to apply it in different laboratories and on different instruments. Here, the original training data has been collected in different laboratories and on the instruments from different vendors, while the log*IE* values for OH-PCBs have been collected in a different lab and different instrument then any of the training data. We have previously investigated the effect of mass spectrometer type on the ionization efficiency values[26] and on comparability of the quantification results from different laboratories [9].

### Matrix effect

The accuracy of quantification, when the analytical standards are sparsely available, depends on the accuracy of the predictive models and on measurement quality. Extending the scope of ionization efficiency predictive model via retraining is a promising avenue for improving predictive models. The quality of measurements, however, depends on the factors affecting LC/ESI/HRMS signal of the suspects, including the matrix effect. The matrix effect is expected to increase for more complex samples, such as serum samples. To evaluate the impact of matrix effect on the quantification, we evaluated the matrix effect by carrying out sample preparation for three different serum volumes (1.4, 2.0, and 4.0 mL serum). In order to achieve generalizable results, a pooled serum sample was used; however, small variations depending on the sample are likely to occur as the concentration of matrix chemicals depends on the individual from whom the sample has been collected. Each of the extracts was fortified with a mixture of thirteen OH-PCB standards with final concentration between 10^−7^ and 10^−9^ M depending on the analyte. The analytes were chosen so that isomeric OH-PCBs did not co-elute, which would have disabled determination of matrix effect for individual isomers. All samples were injected with a volume of 5 or 10 µL.

**Fig. 3:**
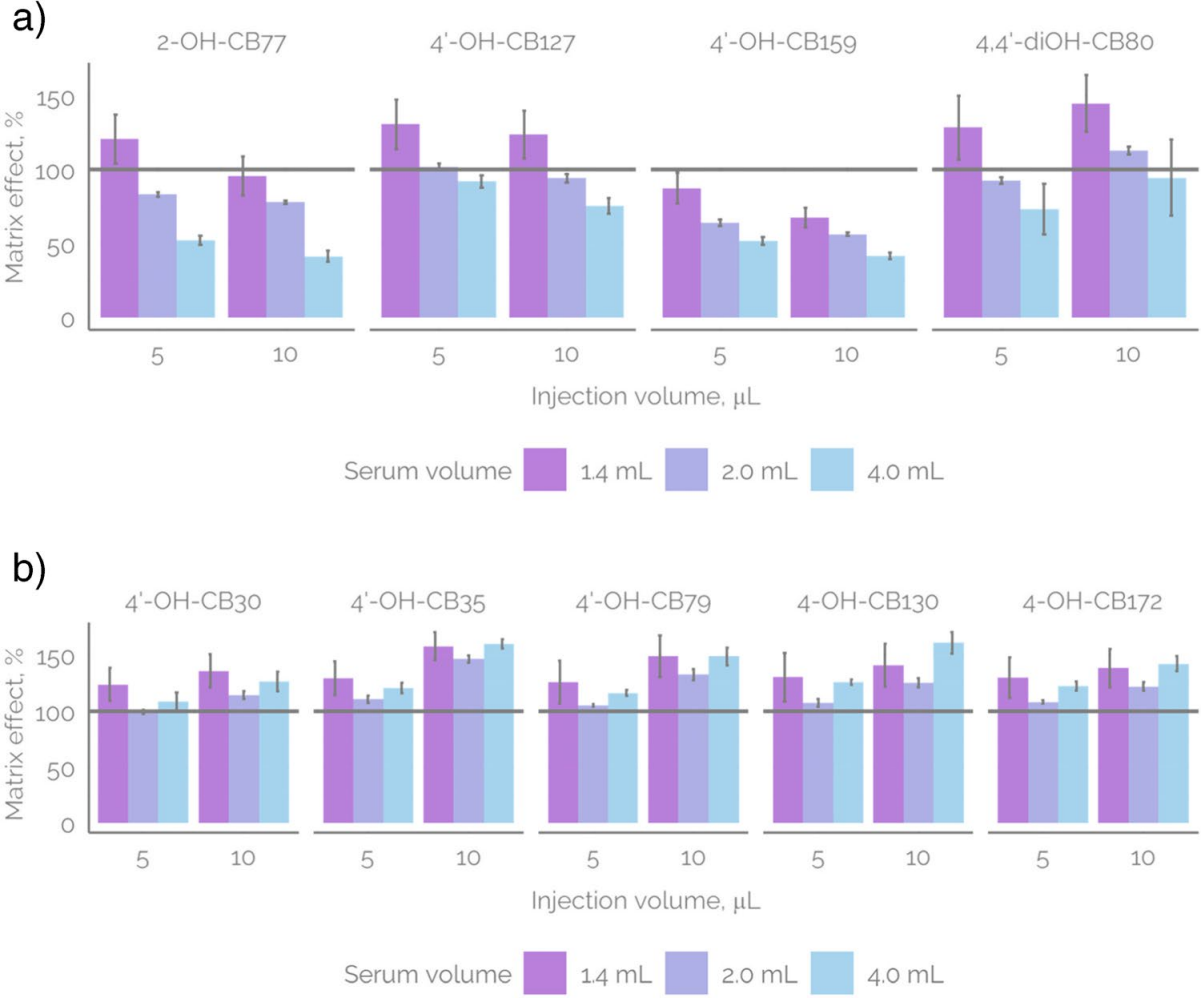
The matrix effect in LC/ESI/HRMS for studied OH-PCBs depending **a** significantly on the serum volume taken for extraction (panels of 1.4 mL, 2 mL, and 4 mL) and **b** significantly on injection volume (5 µL, 10 µL). 2-OH-CB77, 4′-OH-CB159, and 4,4′-diOH-CB80, shown in panel **a**, showed statistically significant variability with both serum volume and injection volume

The matrix effect varied between 37 and 165% (Fig. 3), where 37% indicates a decrease in detected concentration by 63% compared to the spiked concentration due to ionization suppression and 165% indicates a 65% higher detected concentration then the spiked concentration due to ionization enhancement. Analysis of variance (ANOVA) at 95% confidence revealed that for four compounds the volume of sample matrix has a statistically significant impact on the matrix effect (see Fig. 3a). For three out of four of these compounds, the matrix effect showed slight ionization enhancement at the lowest sample matrix volume. At higher sample volumes (2.0 and 4.0 mL), an increasing ionization suppression was observed. This is expected as the concentration of co-eluting matrix components increases with increasing sample volume and this also increases the competition between the analyte and matrix components for the surface charge in ESI [27].

Additionally, for eight compounds, statistically significant variations in the matrix effect were observed with changes in the injection volume. In five of these cases, a slight ionization enhancement was observed under all conditions (Fig. 3b), and the enhancement increased with injection volume. Generally, the possible causes regarding ionization enhancement are not known [11]. For three of the OH-PCBs showing ionization suppression with sample volumes, the suppression also increased with the injection volume (2-OH-CB77, 4′-OH-CB127, and 4′-OH-CB159). This tendency is expected to be facilitated by the competition of the analyte and matrix components for the surface charge of the ESI droplets.

Previously, different strategies have been employed to overcome matrix effect. One of the strategies simply suggests diluting the sample[12] while another strategy suggests consequent dilution of the sample until either no further increase in calculated analyte concentration is observed (indicating overcoming ionization suppression) or extrapolating the calculated analyte concentration to infinite dilution factor if matrix effect cannot be overcome [13]. Here, we observe that some reduction in the matrix effect can also be achieved by using lower injection volumes, which is an easy and fast solution. However, care needs to be taken to not increase the limit of quantification beyond the concentration of analyte present in the sample.

Another way to avoid matrix effects is to improve the removal of the matrix during the sample pretreatment. The sample preparation for the matrix effect study was based on a method designed to analyse OH-PCBs with gas chromatography (GC)/MS [18]. However, the procedure proposed by Hovander also included derivatizion of the phenols and the matrix (mainly lipids) removed with a destructive sulphuric acid treatment. In this study, no further cleanup was performed on the extract after the separation of the OH-PCB from the neutral fraction, which contain the majority of the lipophilic triglycerides and phospholipids. Although achieving a sample extract totally free from matrix is almost impossible, an additional cleanup step suited for the OH-PCBs could be added to optimize the analytical performance.

### Impact of matrix effect on quantification

It was also of interest to evaluate the interplay of prediction accuracy and matrix effect on the quantification accuracy. For this purpose, serum samples spiked with the mix of OH-PCBs were used and the quantification was evaluated for the three OH-PCBs reserved for the test set, namely, 4-OH-CB130, 4-OH-CB107, and 4′-OH-CB30. On average, the concentration prediction errors were 2.0x, 2.5x, and 3.6x (see Table S4). The corresponding prediction errors for neat standard solutions were 2.6x, 2.9x, and 3.1x. However, for all of the compounds, the concentration prediction error varied somewhat with the concentration, matrix concentration, and sample injection volume. For example, in case of 4′-OH-CB30, the lowest error was 3.1x and the highest 4.4x. The latter also being the largest error observed in the whole dataset and though statistically significant from the errors for neat standard solutions, the differences are minimal in practice.

For 4′-OH-CB30, the concentration prediction error is higher in spiked serum sample than in neat standard solution. In case of 4-OH-CB130 and 4-OH-CB107, the mean prediction errors are lower for serum samples than for the standard solutions. This arises from the fact that the model is somewhat overpredicting the ionization efficiency and therefore response factor of 4-OH-CB130 and 4-OH-CB107, which yields under predicted concentrations. However, in serum samples, a slight ionization enhancement is observed and these competing effects somewhat balance out each other. On the other hand, in case of 4′-OH-CB30, the response factor is underpredicted. Alongside this, matrix enhancement occurs for 4′-OH-CB30, increasing the prediction errors further to even larger overestimation. Still, the magnitude of the mean prediction errors depended very little on the serum matrix volume.

All in all, the prediction errors for the serum samples were reasonable and limited primarily by the log*IE* prediction accuracy. The low impact of matrix effect on the prediction accuracy indicates that for wide applicability of quantification in non-targeted and suspect screening the first task is to develop and improve quantification models and the coverage of the chemical space. Combating matrix effect follows as a secondary challenge.

## Conclusions

Here we have shown that ionization efficiency-based quantification can be easily extended for new compound classes by adding a proportion of compounds similar to the analytes of interest to the training data. However, if the similarity of analytes and training compounds is low, so is the prediction accuracy for the ionization efficiency-based quantification. Additionally, we evaluate the impact of electrospray matrix effect on the quantification accuracy and show for the first time that ionization effiency-based quantification can enable quantification of exposomics samples, such as serum samples, without the loss of prediction accuracy.

## Supplementary Information

Below is the link to the electronic supplementary material.

Supplementary file1 (PDF 289 KB)
