# Supplementary material for: Estimation of the concentrations of hydroxylated polychlorinated biphenyls in human serum using ionization efficiency prediction for electrospray
Source: Anal Bioanal Chem. 2022 May 4;414(25):7451–60. doi: 10.1007/s00216-022-04096-2 (PMC9482908; doi:10.1007/s00216-022-04096-2)
Supplement: Supplementary file 1 — Supplementary file1 (PDF 289 KB) [file 216_2022_4096_MOESM1_ESM.pdf]

Supplementary Information

Estimation of the concentrations of hydroxylated polychlorinated biphenyls in human serum using ionization efficiency prediction for electrospray

Sara Khabazbashi,[a] Josefin Engelhardt,[b] Claudia Möckel, [a] Jana Weiss,[b] Anneli Kruve [a,b]

[a] Svante Arrhenius väg 16, Stockholm 106 91, Department of Materials and Environmental Science, Stockholm University

[b] Svante Arrhenius väg 8, Stockholm 106 91, Department of Environmental Science, Stockholm University

Sample preparation

In short, 50 mL human serum from 2 Swedish blood donors were combined in a beaker to give a pooled sample. The serum was transferred to a separation funnel and denatured with 10 mL 6 M hydrochloric acid and 60 mL isopropanol. 60 mL 1:1 isohexane/MTBE was added and shaken for 5 min. The sample was left overnight for maximum separation. The top organic phase was removed to another separation funnel and the aqueous phase was reextracted two more times, allowing 10-15 min for separation each time. First with 50 mL 1:1 isohexane/MTBE and then with 25 mL isohexane. The organic phases were pooled and washed with 40 mL 1% KCl, and then transferred to a round bottom flask. The organic phase was evaporated until approximately 10-15 mL was left and then transferred to a new separation funnel. The residues were rinsed with 15 mL isohexane and added to the separation funnel.

To separate the neutral and polar fraction, 10 mL KOH 1 M was added to the funnel and inverted 30 times. The phases were allowed to separate and the KOH fraction was collected. Reextraction was performed with an additional 2.5 mL KOH 1 M and the funnel inverted 30 times again before allowing separation. Once separated, the KOH phase was once again collected. The two alkaline solutions were pooled and 4 mL HCl 2 M was added to protonate the hydroxyl groups. The funnel was inverted a couple of times and then phenolic compounds were extracted with three washes of 9:1 isohexane/MTBE 15 mL, 5mL x 2. The pooled organic phases were transferred to a beaker.

The sample was then divided into aliquots corresponding to 1.4 mL, 2 mL and 4 mL serum in triplicates. The procedure was duplicated in order to have identical setup for samples fortified with the native OH-PCBs for quality control. The extracts were evaporated under a gentle stream of nitrogen gas to approximately 50 µL to remove residual isohexane/MTBE. Half of the samples were fortified with 100 µL of the standard mixtures (31 to 59 ng/mL). The equivalent amount methanol was added to the other samples. All samples were finally evaporated to 100 µL before injection on the LC-MS system.

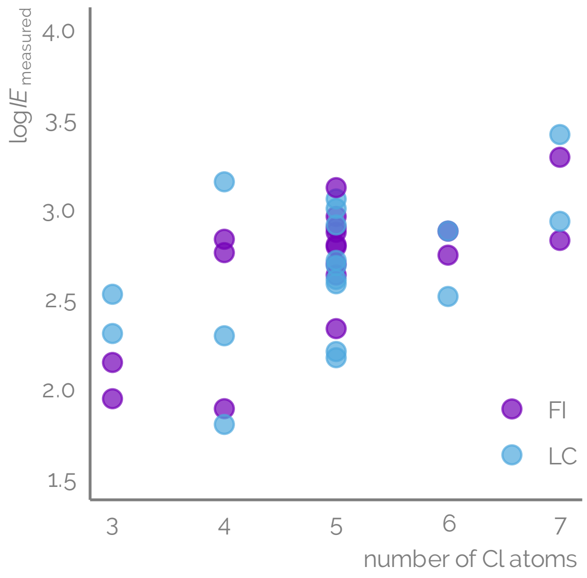

Figure S1 The increase in log*IE* with the increasing number of chlorine atoms. Pink points indicate flow injection (FI) analysis where the organic modifier content was kept at 80% while grey points refer to liquid chromatography (LC) measurements where the organic modifier content depends on the retention time of the compound.

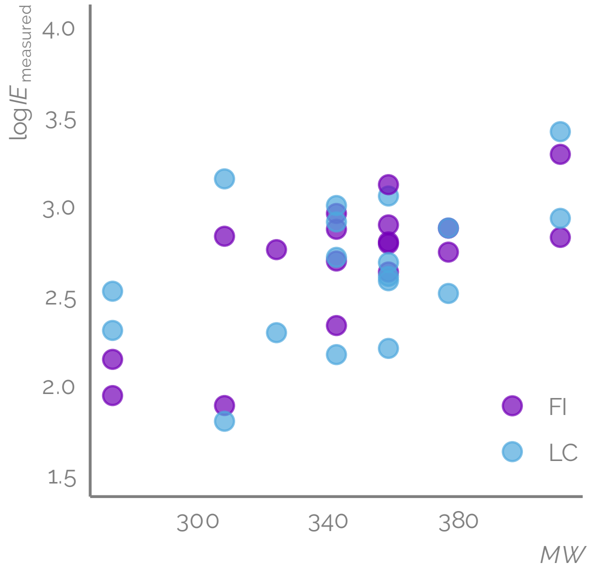

Figure S2 The increase in log*IE* with the increasing molecular weight. Pink points indicate flow injection (FI) analysis where the organic modifier content was kept at 80% while grey points refer to liquid chromatography (LC) measurements where the organic modifier content depends on the retention time of the compound.

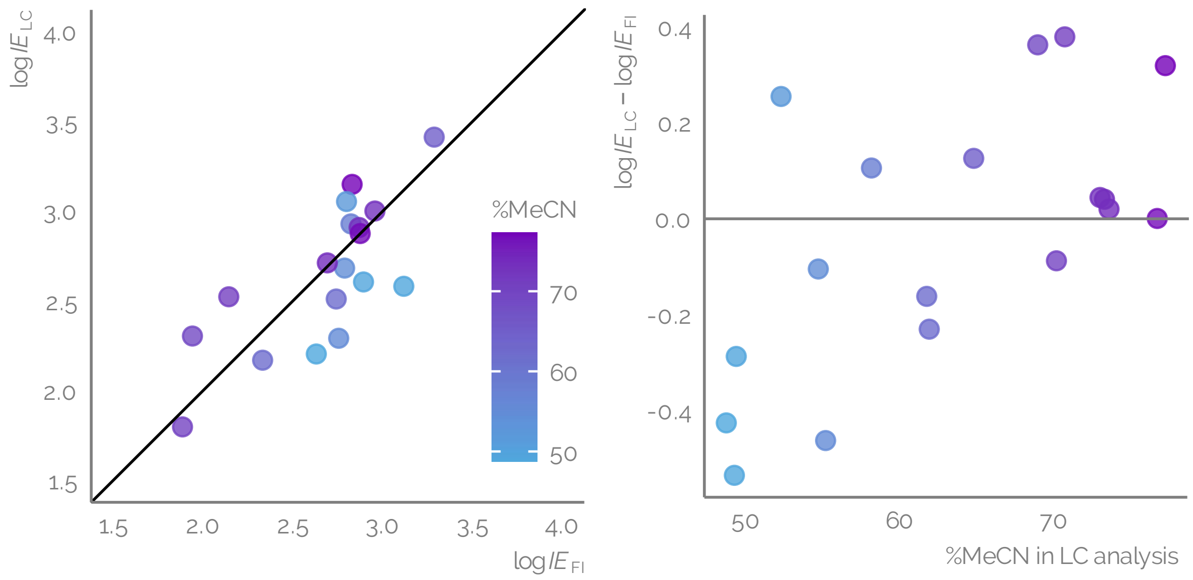

Figure S3 The correlation (R^2 of 0.56) between log*IE* values measured with liquid chromatographic analysis vs flow injection analysis.

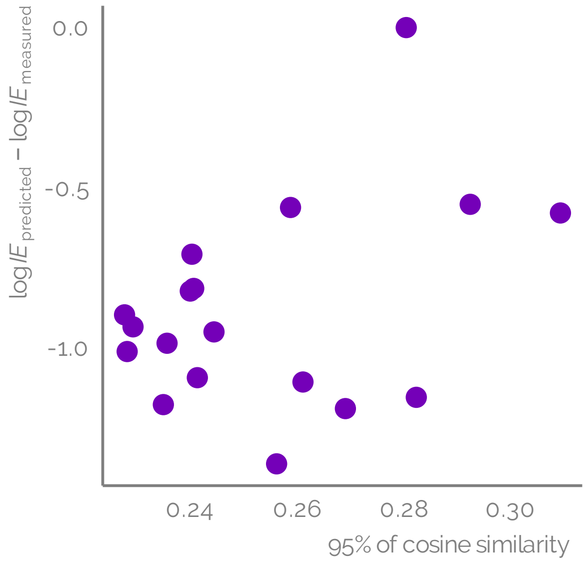

Figure S4 The relationship between the prediction error of the previously trained model by Liigand et al.[1] and the 95th percentile of the cosine similarity to the training data.

Table S1 The measured log*IE* values for all of the OH-PCBs in both trainign and test set measured both with LC and FIA.

		**# of Cl**	**# of OH**	**short name**	**long name**	**SMILES**	**%MeCN**	**log*IE***
Training set	FI	3	1	4'-OH-CB35	4-OH-3,3',4'-trichlorobiphenyl	OC(C(Cl)=C1)=CC=C1C2=CC(Cl)=C(Cl)C=C2	80.0	2.14
		4	1	4'-OH-CB79	4-OH-3,3',4',5-tetrachlorobiphenyl	ClC1=C(Cl)C=C(C2=CC(Cl)=C(O)C(Cl)=C2)C=C1	80.0	1.88
				2-OH-CB77	2-OH-3,3',4,4'-tetrachlorobiphenyl	ClC1=CC=C(C2=C(O)C(Cl)=C(Cl)C=C2)C=C1Cl	80.0	2.83
			2	4,4'-diOH-CB80	4,4'-diOH-3,3',5,5'-tetrachlorobiphenyl	OC1=C(Cl)C=C(C2=CC(Cl)=C(O)C(Cl)=C2)C=C1Cl	80.0	2.76
		5	1	4'-OH-CB120	4-OH-2',3,4',5,5'-pentachlorobiphenyl	ClC1=CC(Cl)=C(Cl)C=C1C2=CC(Cl)=C(O)C(Cl)=C2	80.0	2.69
				4-OH-CB108	4-OH-2',3,3',4',5-pentachlorobiphenyl	ClC1=C(Cl)C(Cl)=C(C2=CC(Cl)=C(O)C(Cl)=C2)C=C1	80.0	2.96
				4'-OH-CB127	4-OH-3,3',4',5',5-pentachlorobiphenyl	OC(C(Cl)=C1)=C(Cl)C=C1C2=CC(Cl)=C(Cl)C(Cl)=C2	80.0	2.87
			2	4,4'-diOH-CB83	4,4'-diOH-2,2',3,3',5-pentachlorobiphenyl	ClC1=C(O)C(Cl)=C(Cl)C(C2=C(Cl)C(Cl)=C(O)C=C2)=C1	80.0	2.89
				4,2'-diOH-CB107	4,2'-diOH-2,3,3',4',5-pentachlorobiphenyl	ClC1=C(Cl)C(O)=C(C2=CC(Cl)=C(O)C(Cl)=C2Cl)C=C1	80.0	2.79
				4,3'-diOH-CB90	4,3'-diOH-2,2',3,4',5-pentachlorobiphenyl	ClC1=C(O)C(Cl)=C(C2=CC(Cl)=C(O)C(Cl)=C2Cl)C=C1	80.0	2.63
				4,3'-diOH-CB107	4,3'-diOH-2,3,4',5,5'-pentachlorobiphenyl	OC(C(Cl)=C1Cl)=C(Cl)C=C1C2=CC(O)=C(Cl)C(Cl)=C2	80.0	2.80
		6	1	4'-OH-CB159	4-OH-2',3,3',4',5,5'-hexachlorobiphenyl	OC(C(Cl)=C1)=C(Cl)C=C1C2=CC(Cl)=C(Cl)C(Cl)=C2Cl	80.0	2.88
		7	1	4-OH-CB172	4-OH-2,2',3,3',4',5,5'-heptachlorobiphenyl	ClC1=C(Cl)C=C(C2=C(Cl)C(Cl)=C(O)C(Cl)=C2)C(Cl)=C1Cl	80.0	3.29
			1	4-OH-CB193	4-OH-2,3,3',4',5,5',6-heptachlorobiphenyl	ClC1=C(Cl)C=C(C2=C(Cl)C(Cl)=C(O)C(Cl)=C2Cl)C=C1Cl	80.0	2.82
	LC	3	1	4'-OH-CB35	4-OH-3,3',4'-trichlorobiphenyl	OC(C(Cl)=C1)=CC=C1C2=CC(Cl)=C(Cl)C=C2	70.8	2.52
		4	1	2-OH-CB77	2-OH-3,3',4,4'-tetrachlorobiphenyl	ClC1=CC=C(C2=C(O)C(Cl)=C(Cl)C=C2)C=C1Cl	77.3	3.15
				4'-OH-CB79	4-OH-3,3',4',5-tetrachlorobiphenyl	ClC1=C(Cl)C=C(C2=CC(Cl)=C(O)C(Cl)=C2)C=C1	70.2	1.80
			2	4,4'-diOH-CB80	4,4'-diOH-3,3',5,5'-tetrachlorobiphenyl	OC1=C(Cl)C=C(C2=CC(Cl)=C(O)C(Cl)=C2)C=C1Cl	55.2	2.29
		5	1	4'-OH-CB120	4-OH-2',3,4',5,5'-pentachlorobiphenyl	ClC1=CC(Cl)=C(Cl)C=C1C2=CC(Cl)=C(O)C(Cl)=C2	73.6	2.71
				4-OH-CB108	4-OH-2',3,3',4',5-pentachlorobiphenyl	ClC1=C(Cl)C(Cl)=C(C2=CC(Cl)=C(O)C(Cl)=C2)C=C1	73.1	3.00
				4'-OH-CB127	4-OH-3,3',4',5',5-pentachlorobiphenyl	OC(C(Cl)=C1)=C(Cl)C=C1C2=CC(Cl)=C(Cl)C(Cl)=C2	73.4	2.91
			2	4,4'-diOH-CB83	4,4'-diOH-2,2',3,3',5-pentachlorobiphenyl	ClC1=C(O)C(Cl)=C(Cl)C(C2=C(Cl)C(Cl)=C(O)C=C2)=C1	49.4	2.61
				4,2'-diOH-CB107	4,2'-diOH-2,3,3',4',5-pentachlorobiphenyl	ClC1=C(Cl)C(O)=C(C2=CC(Cl)=C(O)C(Cl)=C2Cl)C=C1	54.7	2.68
				4,3'-diOH-CB107	4,3'-diOH-2,3,4',5,5'-pentachlorobiphenyl	OC(C(Cl)=C1Cl)=C(Cl)C=C1C2=CC(O)=C(Cl)C(Cl)=C2	52.3	3.05
				4,3'-diOH-CB90	4,3'-diOH-2,2',3,4',5-pentachlorobiphenyl	ClC1=C(O)C(Cl)=C(C2=CC(Cl)=C(O)C(Cl)=C2Cl)C=C1	48.8	2.20
		6	1	4'-OH-CB159	4-OH-2',3,3',4',5,5'-hexachlorobiphenyl	OC(C(Cl)=C1)=C(Cl)C=C1C2=CC(Cl)=C(Cl)C(Cl)=C2Cl	76.8	2.88
		7	1	4-OH-CB172	4-OH-2,2',3,3',4',5,5'-heptachlorobiphenyl	ClC1=C(Cl)C=C(C2=C(Cl)C(Cl)=C(O)C(Cl)=C2)C(Cl)=C1Cl	64.8	3.41

			1	4-OH-CB193	4-OH-2,3,3',4',5,5',6-heptachlorobiphenyl	ClC1=C(Cl)C=C(C2=C(Cl)C(Cl)=C(O)C(Cl)=C2Cl)C=C1Cl	58.2	2.93
Test set	FI	3	1	4'-OH-CB30	4-OH-2',4',6'-trichlorobiphenyl	OC1=CC=C(C2=C(Cl)C=C(Cl)C=C2Cl)C=C1	80.0	1.94
		5	1	4-OH-CB107	4-OH-2,3,3',4',5-pentachlorobiphenyl	ClC1=C(Cl)C(O)=C(Cl)C=C1C2=CC(Cl)=C(Cl)C=C2	80.0	2.33
		5	2	4,4'-diOH-CB111	4,4'-diOH-2,3,3',5,5'-pentachlorobiphenyl	OC1=C(Cl)C=C(C2=CC(Cl)=C(O)C(Cl)=C2Cl)C=C1Cl	80.0	3.12
		6	1	4-OH-CB130	4-OH-2,2',3,3',4',5-Hexachlorobiphenyl	ClC1=C(Cl)C(Cl)=C(C2=CC(Cl)=C(O)C(Cl)=C2Cl)C=C1	80.0	2.74
	LC	3	1	4'-OH-CB30	4-OH-2',4',6'-trichlorobiphenyl	OC1=CC=C(C2=C(Cl)C=C(Cl)C=C2Cl)C=C1	69.0	2.30
		5	1	4-OH-CB107	4-OH-2,3,3',4',5-pentachlorobiphenyl	ClC1=C(Cl)C(O)=C(Cl)C=C1C2=CC(Cl)=C(Cl)C=C2	61.8	2.17
		5	2	4,4'-diOH-CB111	4,4'-diOH-2,3,3',5,5'-pentachlorobiphenyl	OC1=C(Cl)C=C(C2=CC(Cl)=C(O)C(Cl)=C2Cl)C=C1Cl	49.3	2.58
		6	1	4-OH-CB130	4-OH-2,2',3,3',4',5-Hexachlorobiphenyl	ClC1=C(Cl)C(Cl)=C(C2=CC(Cl)=C(O)C(Cl)=C2Cl)C=C1	62.0	2.51

Table S2 Variable importance for the previously trained random forest model from Liigand et al.

feature	scaled variable importance
SM1_Dzm	100.0
GGI4	67.2
ATS0m	53.4
DELS2	45.7
ETA_Eta_R	35.4
pH.aq.	31.3
SM1_Dze	30.9
ATS4m	29.9
viscosity	16.4
MIC3	13.5
surface_tension	13.3
ATSC0s	12.2
polarity_index	12.1
VR1_Dze	11.0
SpAD_Dzp	8.0
ATS1s	7.7
ATS0s	6.5
WPATH	6.3
NH4	5.5
ZMIC3	5.3

Table S3 Variable importance for the gradient boosted regression trees model trained here.

feature	scaled variable importance
`VPC-6`	100.0
SpMax8_Bhm	47.4
AATS5v	42.8
ATS0m	38.3
AATS2s	36.4
pH.aq.	20.4
viscosity	13.0
surface_tension	12.0
AATS6v	10.3
AATS6i	9.8
ZMIC5	8.4
ETA_dEpsilon_B	8.0
additive_concentration_mM	8.0
`BCUTw-1h`	7.2
polarity_index	7.2
ATS5s	6.4
VR3_Dzs	4.7
AATS6e	3.4
GATS8p	3.3
ETA_EtaP	2.9

Table S4 The prediction error for three OH-PCBs from the test set.

Analyte	logIE_{pred}	$slope_{pred}$ $(mmol)^{-1}$	Sampe (mL)	Inj. vol. (µL)	RT (min)	n (mol)	Area	n_{pred} (mol)	c_{pred} (M)	Error in folds
4-OH-CB130 (4-OH-2,2',3,3',4',5-hexachlorobiphenyl)	2.95	9.63E+09	No matrix	5	11.66	8.42E-14	1.60E+03	3.30E-14	6.60E-09	2.54
			No matrix	10	11.66		3.09E+03	3.20E-14	3.20E-09	2.63
			1.4	5	11.66		2.38E+03	4.90E-14	9.90E-09	1.7
			1.4	5	11.67		2.17E+03	4.50E-14	9.00E-09	1.87
			1.4	5	11.66		1.70E+03	3.50E-14	7.10E-09	2.38
			1.4	10	11.65		4.85E+03	5.00E-14	5.00E-09	1.67
			1.4	10	11.65		4.52E+03	4.70E-14	4.70E-09	1.79
			1.4	10	11.65		3.70E+03	3.80E-14	3.80E-09	2.19
			2	5	11.65		1.78E+03	3.70E-14	7.40E-09	2.28
			2	5	11.66		1.71E+03	3.50E-14	7.10E-09	2.38
			2	5	11.66		1.67E+03	3.50E-14	6.90E-09	2.42
			2	10	11.64		3.87E+03	4.00E-14	4.00E-09	2.1
			2	10	11.63		3.75E+03	3.90E-14	3.90E-09	2.16
			2	10	11.63		4.00E+03	4.20E-14	4.20E-09	2.03
			4	5	11.65		2.04E+03	4.20E-14	8.50E-09	1.99
			4	5	11.66		2.03E+03	4.20E-14	8.40E-09	2
			4	5	11.65		1.97E+03	4.10E-14	8.20E-09	2.06
			4	10	11.62		4.64E+03	4.80E-14	4.80E-09	1.75
			4	10	11.64		5.20E+03	5.40E-14	5.40E-09	1.56
			4	10	11.63		5.09E+03	5.30E-14	5.30E-09	1.59

4-OH-CB107 (4-OH-2,3,3',4',5-pentachlorobiphenyl)	2.72	5.15E+09	No matrix	5	11.66	9.26E-14	8.32E+02	3.20E-14	6.50E-09	2.87
			No matrix	10	11.66		1.59E+03	3.10E-14	3.10E-09	3
			s4a	5	11.65		1.12E+03	4.30E-14	8.70E-09	2.14
			s4b	5	11.65		1.02E+03	4.00E-14	8.00E-09	2.33
			s4c	5	11.65		8.03E+02	3.10E-14	6.20E-09	2.97
			s4a	10	11.64		2.20E+03	4.30E-14	4.30E-09	2.17
			s4b	10	11.64		2.11E+03	4.10E-14	4.10E-09	2.26
			s4c	10	11.63		1.69E+03	3.30E-14	3.30E-09	2.82
			s8a	5	11.65		9.27E+02	3.60E-14	7.20E-09	2.57
			s8b	5	11.65		8.67E+02	3.40E-14	6.70E-09	2.75
			s8c	5	11.65		8.45E+02	3.30E-14	6.60E-09	2.82
			s8a	10	11.63		1.83E+03	3.60E-14	3.60E-09	2.6
			s8b	10	11.62		1.83E+03	3.60E-14	3.60E-09	2.61
			s8c	10	11.62		1.91E+03	3.70E-14	3.70E-09	2.49
			s12a	5	11.64		8.87E+02	3.40E-14	6.90E-09	2.69
			s12b	5	11.66		9.01E+02	3.50E-14	7.00E-09	2.65
			s12c	5	11.64		8.63E+02	3.40E-14	6.70E-09	2.76
			s12a	10	11.6		2.00E+03	3.90E-14	3.90E-09	2.38
			s12b	10	11.63		2.15E+03	4.20E-14	4.20E-09	2.22
			s12c	10	11.62		2.12E+03	4.10E-14	4.10E-09	2.25

4’-OH-CB30<br>(4-OH-2',4',6'-trichlorobiphenyl)	2.12	9.68E+08	No matrix	5	12.94	1.16E-13	1.78E+03	3.70E-13	7.40E-08	3.18
			No matrix	10	12.92		3.36E+03	3.50E-13	3.50E-08	2.99
			s4a	5	12.94		2.42E+03	5.00E-13	1.00E-07	4.32
			s4b	5	12.94		2.29E+03	4.70E-13	9.50E-08	4.08
			s4c	5	12.94		1.91E+03	3.90E-13	7.90E-08	3.4
			s4a	10	12.92		4.94E+03	5.10E-13	5.10E-08	4.4
			s4b	10	12.9		4.75E+03	4.90E-13	4.90E-08	4.23
			s4c	10	12.9		4.01E+03	4.10E-13	4.10E-08	3.57
			s8a	5	12.93		1.81E+03	3.70E-13	7.50E-08	3.22
			s8b	5	12.93		1.76E+03	3.60E-13	7.30E-08	3.13
			s8c	5	12.93		1.75E+03	3.60E-13	7.20E-08	3.12
			s8a	10	12.93		3.86E+03	4.00E-13	4.00E-08	3.44
			s8b	10	12.89		3.71E+03	3.80E-13	3.80E-08	3.3
			s8c	10	12.89		3.93E+03	4.10E-13	4.10E-08	3.5
			s12a	5	12.93		1.87E+03	3.90E-13	7.70E-08	3.32
			s12b	5	12.94		2.10E+03	4.30E-13	8.70E-08	3.74
			s12c	5	12.94		1.84E+03	3.80E-13	7.60E-08	3.28
			s12a	10	12.89		3.94E+03	4.10E-13	4.10E-08	3.51
			s12b	10	12.93		4.51E+03	4.70E-13	4.70E-08	4.02
			s12c	10	12.93		4.30E+03	4.40E-13	4.40E-08	3.83
